# Protection of Spleen Tissue of γ-ray Irradiated Mice against Immunosuppressive and Oxidative Effects of Radiation by Adenosine 5′-Monophosphate

**DOI:** 10.3390/ijms19051273

**Published:** 2018-04-24

**Authors:** Cuilin Cheng, Juanjuan Yi, Rongchun Wang, Li Cheng, Zhenyu Wang, Weihong Lu

**Affiliations:** 1 Food Science and Engineering, School of Chemical Engineering & Technology, Harbin Institute of Technology, Harbin 150090, China; ccuilin@hit.edu.cn (C.C.); yjj2017@zzu.edu.cn (J.Y.); wangrongchun@hit.edu.cn (R.W.); 2 Institute of Extreme Environmental Nutrition and Protection, Harbin Institute of Technology, Harbin 150090, China; 3 School of Life Sciences, Zhengzhou University, Zhengzhou 450001, China; 4 School of Life Science, Heilongjiang University, Harbin 150080, China; chengli@hlju.edu.cn

**Keywords:** 5′-AMP, radioprotection, immuno-regulation, antioxidant activities

## Abstract

The immune system is very sensitive to radiation. This study revealed that adenosine 5′-monophosphate (5′-AMP) increased the DNA contents of the spleen and the spleen index of irradiated mice. Moreover, the exogenous 5′-AMP could significantly repair the ultra-structure of the damaged spleen through transmission electron microscopy. When indicators of the mouse immune system were assessed, the flow cytometry and enzyme-linked immunosorbent assay (ELISA) revealed that the administration of exogenous 5′-AMP could reduce the apoptosis in the splenic cells. It could also regulate the transition of cells towards S phase, increase the proportion of CD4^+^ and CD8^+^ cellular subsets, and enhance the secretion of interleukin-2 (IL-2), IL-4, IL-10, and interferon-γ (IFN-γ). These effects were associated with a decrease in oxidative stress, as evidenced by changes in superoxide dismutase (SOD), glutathione peroxidase (GSH-Px), catalase (CAT), reduced glutathione (GSH), and malondialdehyde (MDA) levels of spleen tissues. These results suggested that exogenous 5′-AMP could repair the damaged spleen, increase the spleen index, and regulate the cell cycles and apoptosis. There was an increase in the production of various cytokines and play a protective role on the immune system of irradiated mice by dynamically adjusting the REDOX balance.

## 1. Introduction

Nucleotides, as basic units of nucleic acids, are a class of low molecular weight biological compounds. They play key roles in numerous biological processes, such as involving in the synthesis of DNA and RNA, encoding and decoding genetic information, providing cellular energy, influencing protein bio-synthesis and modulating the expressions of a number of genes [1,2]. Nucleotides are synthesized de novo in most tissues, but some immune cells lack this process. The exogenous supply is used to prove the necessity for immune regulation, particularly under certain conditions, such as when the body is in rapid growth or suffers from an immunological challenge, intestinal injury, or liver dysfunction [3,4,5,6]. Indeed, Hu G. et al. have discussed the regulatory effects of dietary nucleotides on immune responses [7], and several studies have also confirmed that they work through increasing lymphocyte proliferation, plasma lysozyme activity, and leukocyte stimulated with concanavalin (ConA) and phytohamagglutinin (PHA-P) [8,9,10,11]; stimulating superoxide anion and total serum protein productions [12]; enhancing the natural killer cell activity, macrophage activation and phagocytosis and delayed hypersensitivity. The productions of immunoglobulin A (IgA) and IL-1β and the genetic expressions of interleukin-6 (IL-6), IL-8, Toll-like receptor (TLR-9), TLR-4, and Tollip [13,14]; promoting the popliteal lymph-node cytokine secretions such as interferon-γ (IFN-γ), IL-10, and tumor necrosis factor-α (TNF-α) [15]; and improving superoxide dismutase (SOD) activity and decreasing malondialdehyde (MDA) concentration [16].

However, research to date on dietary nucleotides have mostly focused on a mixture of nucleotides containing adenosine 5′-monophosphate (5′-AMP), rather than individual nucleotide for regulating immunity system except Hossain M.S. et al.’s initial report, in which 5′-AMP supplemented diets could increase significantly total serum protein, lysozyme activity, and agglutination antibody titer in marine fish [17]. Moreover, there is no report on whether 5′-AMP can regularly immune activity even protect an immune system under radiation condition.

Exposure to ionizing radiation (IR) could lead to the damages of the immune organs including the spleens and the thymus damages, and the decline of immunity. These indicate that the immune modulatory system is highly sensitive to IR [18]. Based on the mentioned research on immunomodulatory effects of nucleotides, we propose 5′-AMP might improve immunomodulatory function under IR conditions. Our previous research has shown that the supplement of 5′-AMP could reverse the liver cells DNA damage and reduce the apoptosis levels of liver cells induced by ^60^Co γ-ray radiation [19]. Additionally, it has also been demonstrated that after the administration of 5′-AMP (0.16 g/kgBW/day), the MDA content was significantly reduced. The levels of SOD and GSH-Px were increased in comparison with the IR group in the livers [20]. Therefore, this study was designed to investigate 5′-AMP actions on IR-induced immunosuppression in mice and further help understand the protection mechanisms of 5′-AMP against IR.

## 2. Results

### 2.1. Effect of 5′-AMP on Immune Organ Index

The spleen is the largest immune organ in the body, and its weight can reflect irradiation-induced damage. Here, the experimental mice were grouped according to our previous research with small modification [20]. Mice were divided into six groups including normal group (no treatment), radiation model group (radiation alone), positive control group (radiation + berberine hydrochloride, 20 mg/kgBW/day) and 5′-AMP + irradiation groups at different doses (radiation + 0.08 g/kgBW/day, 0.16 g/kgBW/day and 0.64g/kgBW/day, respectively). Changes in the spleen index between different groups were shown in Table 1. Spleens in the model group were significantly smaller than those in the normal group (0.14 vs. 0.49; *p* < 0.01), suggesting that 4 Gy ^60^Co γ-ray induced damage to the spleen. The spleen index of the positive control group was higher than the model group (0.22 vs. 0.14; *p* < 0.05), and significantly smaller than Group I (0.22 vs. 0.49; *p* < 0.01). After the treatment with 5′-AMP, the spleen index increased compared with Group II. The spleen index of mice treated with the other doses of 5′-AMP was not significantly different from Group III. These results suggested that 5′-AMP intake before radiation could improve the recovery of the spleen against the effects of radiation.

### 2.2. Effect of 5′-AMP on Spleen Ultra-Structure after Whole Body Irradiation of Mice

**Figure 1:**
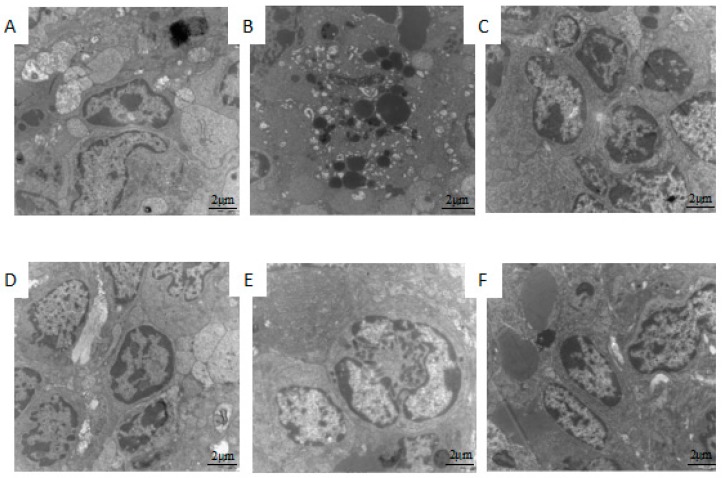
Effect of 5′-AMP on ultra-structure morphology of mouse spleen after γ-ray irradiation (10,000×). (**A**) Group I: Normal group; (**B**) Group II: Model group; (**C**) Group III: Positive control (berberine hydrochloride); (**D**) Group IV: Radiation + 5′-AMP (0.08 g/kgBW/day); (**E**) Group V: Radiation + 5′-AMP (0.16 g/kgBW/day); and (**F**) Group VI: Radiation + 5′-AMP (0.64 g/kgBW/day).

Ultra-structures of the damaged spleens from different groups were presented in Figure 1. It was observed that the untreated mouse splenocytes in the normal group contained round nucleus. In the nucleus, there was abundant euchromatin and one distinct centrally located nucleolus, several round or oval mitochondria, a few free ribosomes, and rough endoplasmic reticulum.

In the model group, there were morphological changes of apoptotic cells such as nucleus chromatin condensation, nuclear fragmentation, nuclear dissolution, and apoptosis body. Rough endoplasmic reticulum was broken and the attached ribosome shed partly. In the positive control group, cellular membrane was intact, most of nuclear membrane were clear and complete. The heterochromatin appeared as a very thin and consistent border around the inner aspect of the nuclear envelope, mitochondria were sound and cristae were unbroken. After treatment with different concentrations of 5′-AMP, apoptotic splenocytes were found to be decreased. The ultra-structure of the mouse spleen in 0.16 g/kgBW/day group recovered clearly. The results revealed that 5′-AMP could reduce the spleen structural damage induced by radiation.

### 2.3. Effects of 5′-AMP on DNA Concentrations in Radiated Mice

**Figure 2:**
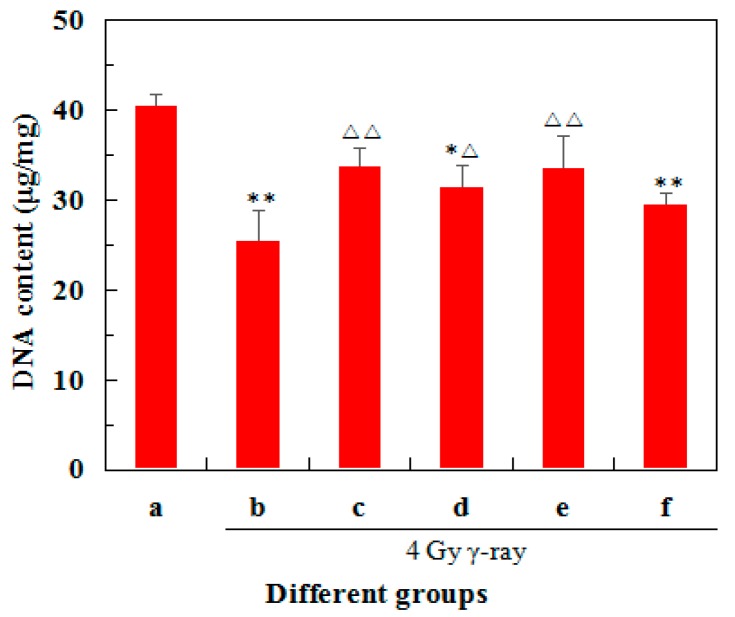
Effects of 5′-AMP on splenic DNA content in mice treated with γ-ray (mean ± SD, *n* = 6). (a) Group I: Normal group; (b) Group II: Model group; (c) Group III: Positive control (berberine hydrochloride); (d) Group IV: Radiation + 5′-AMP (0.08 g/kgBW/day); (e) Group V: Radiation + 5′-AMP (0.16 g/kgBW/day); and (f) Group VI: Radiation + 5′-AMP (0.64 g/kgBW/day). * *p* < 0.05 and ** *p* < 0.01 compared with the normal group; ^Δ^ *p* < 0.05 and ^ΔΔ^ *p* < 0.01 compared with the model group.

Figure 2 showed the effects of 5′-AMP on DNA concentrations in the spleens treated with 4 Gy ^60^Co γ-ray. Compared with the normal group, the model group exhibited a much lower DNA concentration (*p* < 0.01). After the administration of 5′-AMP and berberine hydrochloride, the DNA contents increased significantly (*p* < 0.05) compared with the model group. At a concentration of 0.16 g/kgBW/day, DNA content was much closer to the positive control group and had no significant difference with the normal group (*p* < 0.01). These data suggested that 5′-AMP had a positive effect on the DNA damage of in irradiated mice.

### 2.4. Effects of 5′-AMP on Radiation-Induced Cell-Cycle Arrest in Mouse Splenocytes

**Figure 3:**
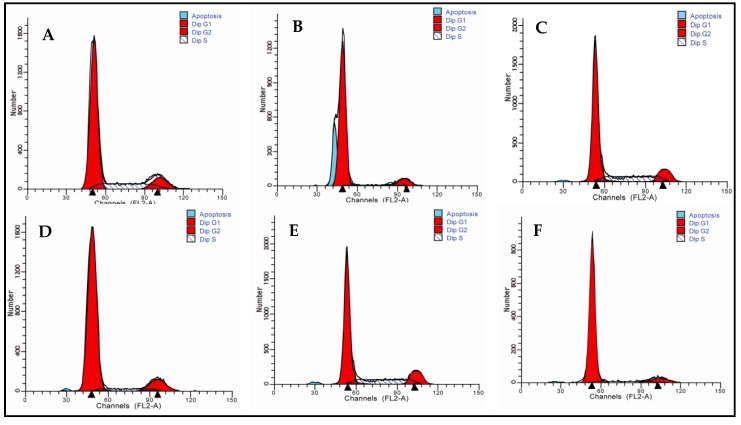

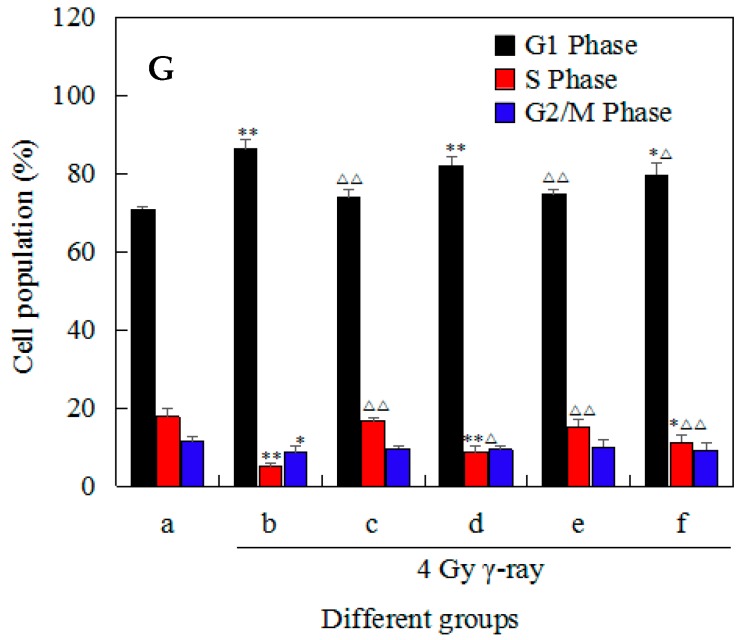
Cell-cycle analysis of splenocytes in different groups (*n* = 6). (**A**) Group I: Normal group; (**B**) Group II: Model group; (**C**) Group III: Positive control (berberine hydrochloride); (**D**) Group IV: Radiation + 5′-AMP (0.08 g/kgBW/day); (**E**) Group V: Radiation + 5′-AMP (0.16 g/kgBW/day); (**F**) Group VI: Radiation + 5′-AMP (0.64 g/kgBW/day); and (**G**) Quantity analysis (mean ± SD, *n* = 6). * *p* < 0.05 and ** *p* < 0.01 compared with the normal group; ^Δ^ *p* < 0.05 and ^ΔΔ^ *p* < 0.01 compared with the model group.

Effects of 5′-AMP on radiation-induced cell-cycle arrest in mouse splenocytes were assessed by flow cytometry. Data in Figure 3 showed the effects of 5′-AMP on the cell cycle phase (G_0_/G_1_, S and G_2_/M) distribution of splenocytes using flow cytometry with propidium iodide (PI) staining. Compared with the normal group (70.91 ± 0.68%), more splenocytes in the model group were arrested in G_0_/G_1_ phase (86.28 ± 2.56%). Whereas the number of splenocytes in S (5.07 ± 0.96%, *p* < 0.01) and G_2_/M phases (8.65 ± 1.75%, *p* < 0.05) decreased significantly. After treatment with different concentrations of 5′-AMP, the number of cells in G_0_/G_1_ decreased, while those in S and G_2_/M phases increased. At the concentration of 0.16 g/kgBW/day, the number of cells in G_0_/G_1_ and S phases were (74.67 ± 1.15%) and (15.25 ± 2.01%). This was comparable to the normal group (*p* > 0.05). Moreover, 5′-AMP did not induce significant changes in the number of cells in G_2_/M phase at the tested concentrations. These results suggested that 5′-AMP could regulate the distribution of splenocytes cycle from irradiated mice by inhibiting cells arrest in the G_0_/G_1_ phase.

### 2.5. Effects of 5′-AMP on Splenocytes Apoptosis Induced by Irradiation

**Figure 4:**
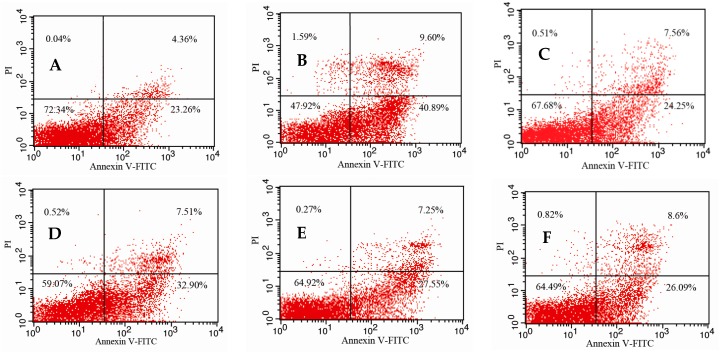

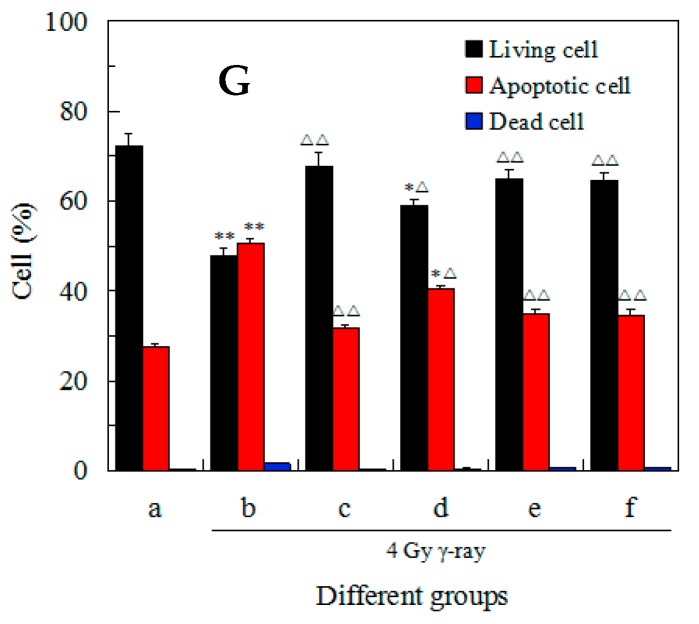
Determination of splenocyte apoptosis in different groups assessed by flow cytometry using the fluorescein isothiocyanate (FITC)-Annexin V/propidium iodide (PI) double staining assay. (**A**) Group I: Normal group; (**B**) Group II: Model group; (**C**) Group III: Positive control (berberine hydrochloride); (**D**) Group IV: Radiation + 5′-AMP (0.08 g/kgBW/day); (**E**) Group V: Radiation + 5′-AMP (0.16 g/kgBW/day); (**F**) Group VI: Radiation + 5′-AMP (0.64g/kgBW/day); and (**G**) Quantity analysis (mean ± SD, *n* = 6). * *p* < 0.05 and ** *p* < 0.01 compared with the normal group; ^Δ^ *p* < 0.05 and ^ΔΔ^ *p* < 0.01 compared with the model group.

Figure 4 showed a contour diagram of fluorescein isothiocyanate (FITC)-Annexin V/PI flow cytometry of splenocytes isolated from different groups and direct comparisons between groups. Compared with the normal group (27.62%), the percentage of apoptotic cells in the model group (50.49%) increased significantly (*p* < 0.01), suggesting that irradiation induced splenocytes damage and apoptosis. After treatment with different concentrations of 5′-AMP, the percentage of apoptotic splenocytes decreased to different degrees. At concentrations of 0.16 g (34.80%) and 0.64 g/kgBW/day (34.69%), the rate of apoptosis was significantly lower than in the model group, and was similar to the positive control group (31.81%). This suggested that 5′-AMP could protect splenocytes against radiation-induced damage by reducing splenocytes apoptosis.

### 2.6. Effects of 5′-AMP on the Changes in Spleen Lymphocyte Subsets after Irradiation

**Figure 5:**
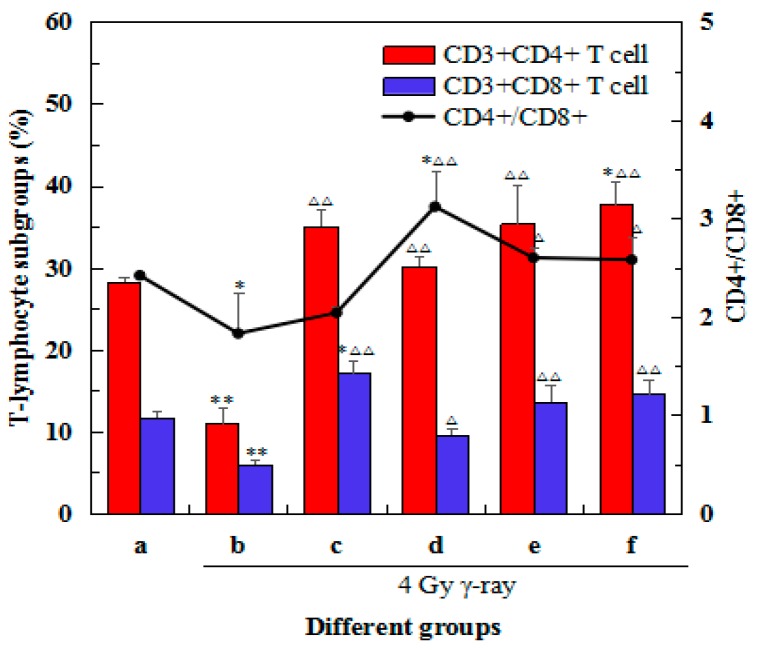
Effects of 5′-AMP on the changes in spleen lymphocyte subsets (CD3^+^CD4^+^ and CD3^+^CD4^+^) after irradiation. (a) Group I: Normal group; (b) Group II: Model group; (c) Group III: Positive control (berberine hydrochloride); (d) Group IV: Radiation + 5′-AMP (0.08 g/kgBW/day); (e) Group V: Radiation + 5′-AMP (0.16 g/kgBW/day); (f) Group VI: Radiation + 5′-AMP (0.64 g/kgBW/day). * *p* < 0.05 and ** *p* < 0.01 compared with the normal group; ^Δ^ *p* < 0.05 and ^ΔΔ^ *p* < 0.01 compared with the model group.

Percentages of different T lymphocyte sub-types in the spleens from different groups were determined and are presented in Figure 5. There was a decrease in the percentages of CD3^+^CD4^+^ and CD3^+^CD8^+^ lymphocytes (*p* < 0.01) after ^60^Co γ-ray (4 Gy). After treatment with different concentrations of 5′-AMP, the percentages of CD3^+^CD4^+^ and CD3^+^CD8^+^ lymphocytes and the ratio of CD4^+^/CD8^+^ T cells increased to different extents. After treatment with 0.16 g/kgBW/day, the percentages of CD3^+^CD4^+^ (35.38%) and CD3^+^CD8^+^ (13.60%) lymphocytes were similar to the positive control group (35.05% and 17.14%, respectively). This was significantly higher than the model group (10.98% and 6.01%, respectively, *p* < 0.01), but comparable to the normal group (28.28% and 11.68%, respectively, *p* < 0.05). Similarly, the ratio of CD4^+^/CD8^+^ T cells in the 5′-AMP 0.16 and 0.64 g/kgBW/day groups were significantly higher than the model group (2.60 and 2.58, respectively, vs. 1.83, *p* < 0.05). These results suggested that 5′-AMP could regulate the distribution of spleen lymphocyte subsets from irradiated mice by changing the percentages of CD3^+^CD4^+^ and CD3^+^CD8^+^ lymphocytes.

### 2.7. Effects of 5′-AMP on the Cytokine Concentrations in Lymphocyte Supernatants after Irradiation

**Figure 6:**
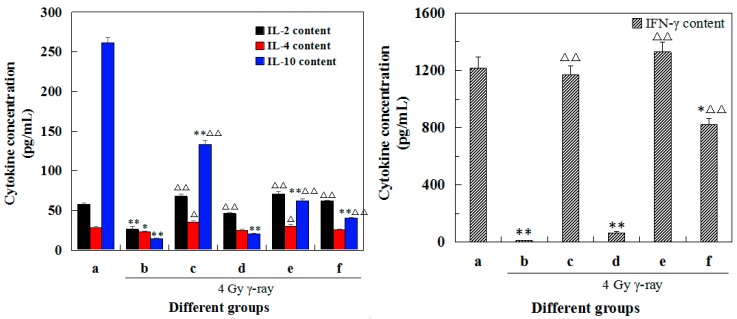
Effects of 5′-AMP on cytokine concentration in the supernatant of lymphocytes from mice treated with γ-ray irradiation (mean ± SD, *n* = 6). (a) Group I: Normal group; (b) Group II: Model group; (c) Group III: Positive control (berberine hydrochloride); (d) Group IV: Radiation + 5′-AMP (0.08 g/kgBW/day); (e) Group V: Radiation + 5′-AMP (0.16 g/kgBW/day); and (f) Group VI: Radiation + 5′-AMP (0.64 g/kgBW/day). * *p* < 0.05 and ** *p* < 0.01 compared with the normal group; ^Δ^ *p* < 0.05 and ^ΔΔ^ *p* < 0.01 compared with the model group.

After observing morphological changes of spleen cells and knowing that 5′-AMP can ameliorate the irradiation-induced abnormal morphology of splenocytes, we further detected the cytokine secretion in culture medium (Figure S1). Cytokine concentrations (IL-2, IL-4, IL-10, and IFN-γ) were calculated based on a standard curve for each enzyme-linked immunosorbent assay (ELISA) plate. The changes in cytokine concentrations in different groups were shown in Figure 6. Comparative to the normal group, the concentration of all cytokines in the model group decreased significantly (*p* < 0.05 or *p* < 0.01). After treatment with different concentrations of 5′-AMP, cytokine concentrations increased compared with the model group to varying degrees. At the concentration of 0.16 g/kgBW/day, IL-2 and IFN-γ concentrations were much closer to the positive control group, and were not significantly different from the normal group. In contrast, IL-10 levels were significantly different from the normal group. IL-4 concentrations were comparable between all groups, except for the positive control group. These results suggested that 5′-AMP could improve splenocytes cytokine secretion and modulate spleen inflammation.

### 2.8. Effects of 5′-AMP on Antioxidant Defence of Spleens in Irradiated Mice

**Figure 7:**
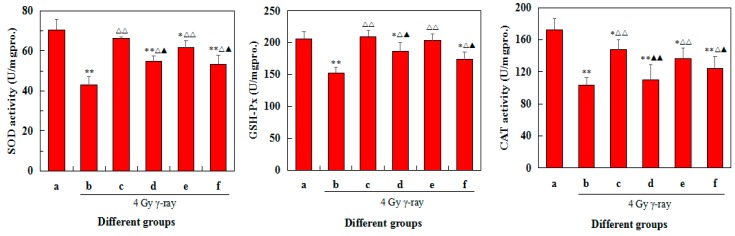

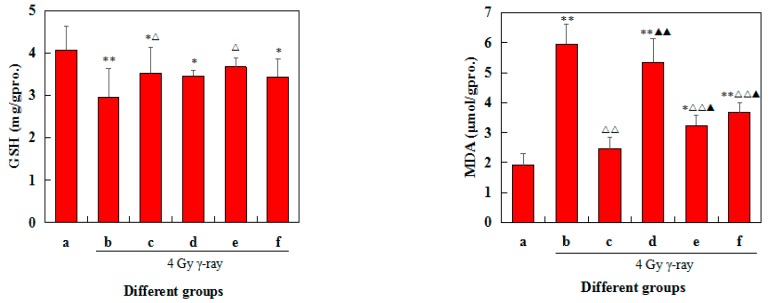
Effects of different doses 5′-AMP pretreatment on the SOD, GSH-Px, CAT, GSH, and MDA levels of spleen tissues (mean ± SD, *n* = 6). (a) Group I: Normal group; (b) Group II: Model group; (c) Group III: Positive control (berberine hydrochloride); (d) Group IV: Radiation + 5′-AMP (0.08 g/kgBW/day); (e) Group V: Radiation + 5′-AMP (0.16 g/kgBW/day); and (f) Group VI: Radiation + 5′-AMP (0.64 g/kgBW/day). * *p* < 0.05 and ** *p* < 0.01 compared with the normal group; ^Δ^ *p* < 0.05 and ^ΔΔ^ *p* < 0.01 compared with the model group. ^▲^ *p* < 0.05 and ^▲▲^ *p* < 0.01 compared with the positive control group.

As shown in Figure 7, ^60^Co γ-ray significantly induced the levels of SOD, GSH-Px, CAT, and GSH and increased MDA content of the radiation group in the spleens (*p* < 0.05 or *p* < 0.01). It follows that 4.0 Gy ^60^Co γ-ray can induce spleen injury.

In contrast, 5′-AMP treatment substantially restored all the indexes equivalent to that of the control group. Among three 5′-AMP doses, 0.16 g/kgBW/day showed the most obvious positive effects in irradiated mice. SOD, GSH-Px, CAT activities, and GSH content significantly increased (*p* < 0.05 or *p* < 0.01), while MDA content substantially decreased (*p* < 0.05 or *p* < 0.01) compared with the radiation group. Moreover, SOD, GSH-Px, CAT, and GSH were recovered similar level of positive group. These data suggest that the protection of 5′-AMP on spleen may be involved in free radical scavenging and MDA production inhibition.

## 3. Discussion

When cells apoptosis was analyzed quantitatively using flow cytometry, a significant decrease in apoptosis was detected after the mice were administered 5′-AMP. Studies on DNA concentrations are increasingly important, since the DNA contents in a tissue is an index for expressing other biochemical contents such as protein contents, and cell division and development. DNA content can reflect the cells proliferation activity, so increased DNA content suggests the activation of metabolic process. In the present study, the DNA content of irradiated spleens in the 5′-AMP groups were increased compared with the model group, which suggested that 5′-AMP increased cell survival and division by inhibiting radiation-induced apoptosis. Previously, Frankič also reported that dietary nucleotides could reduce the amount of DNA damage induced by T-2 toxin in immune cells [3]. In addition, the dietary nucleotides could reduce cyclophosphamide-induced DNA damage in immune organs in mice [21]. Our results were consistent with these reports, since nucleotides exerted protective effects on DNA.

B and T lymphocytes play an important role in adaptive immunity, and are the central cells of the immune system. Once stimulated by certain mitogens, these cells are activated, and enter the cell cycle. Studies have shown that IR can cause cell cycle disorders, such as G_1_ arrest, and S and G_2_ phase delays. This response is thought to be primarily a protective defense mechanism against external damage to ensure genetic stability. G_1_ phase arrest and S phase delay can provide sufficient time for cells to repair damaged DNA. If it is not repaired, DNA damage can result in apoptosis; if it is mis-repaired, it can lead to mutations, genetic instability, and cancer. Therefore, regulating the cells cycle is a way to protect the cell against radiation-induced damage. In our study, the administration of 5′-AMP could regulate the spleen cells cycle effectively in irradiated mice. The number of cells in G_0_/G_1_ decreased, whereas those in S and (M + G_2_) phases increased. Therefore, 5′-AMP likely exerted protective effects against radiation by promoting the entrance of cells into late S phase. Our results are consistent with observations by Rudolph et al. [22], who reported that nucleotide restriction may cause the arrest of T lymphocytes in the G phase of the cells cycle, inhibiting the transition of lymphocytes to S phase to elicit the necessary immunological signals.

T lymphocytes also play an important role in cellular immunity. They exert many biological effects, such as modulating antibody production by B cells, responding to specific antigens and mitogens, and cytokine productions. Subsets of T lymphocytes can be distinguished by the presence of CD4 and CD8 membrane glycoproteins on their surfaces. In general, CD4^+^ cells act as helper cells, and CD8^+^ cells function as cytotoxic cells. The cells can also be separated into two major functional subpopulations (Th1 and Th2 cells) according to the cytokines they secrete. The Th1 response produces cytokines (including IL-2, IFN-γ, and TNF-β) that activate mainly T cells and macrophages, whereas the Th2 response secretes cytokines (including IL-4, IL-6, and IL-10) that activate mainly B cells and immune responses that produce IgE [23,24]. Tc cells, which contain perforin and granzyme, display cytotoxic ability. They can recognize target cells and induce cell lysis or apoptosis.

Studies have confirmed the beneficial effects of nucleotides on improving the Th1 immune response [25,26]. Van Buren et al. proposed that dietary nucleotides exert effects on the immune response by acting on the T helper/inducer population, and predominantly affecting the initial phase of antigen processing and lymphocyte proliferation [27]. After radiation exposure, we observed that the percentage of CD4^+^ and CD8^+^ cells, and the ratio of CD4^+^/CD8^+^, decreased. However, in the three groups of mice treated with 5′-AMP there was an obvious increase in the percentage in these markers, albeit without a dose-dependent relationship. Levels of IFN-γ and IL-2 were also increased significantly in 5′-AMP-treated mice. This suggests that the major effect of 5′-AMP was to activate Th1 cells. Activated Th1 cells secreted IFN-γ and IL-2, which played important roles in activating the proliferation and differentiation of Tc cells (CD8^+^ T cells). IFN-γ levels could also be enhanced by Tc cells. In addition, increased levels of IL-4 and IL-10 were detected in the supernatant of induced lymphocytes, suggesting that 5′-AMP could also induce the Th2 response, which plays an important role in the activation of immunity involving the maintenance of the inflammatory balance. The results suggest 5′-AMP functions as an immune-regulatory molecule, which is consistent with previous results assessing the weight coefficients and apoptosis in the spleen.

**Figure 8:**
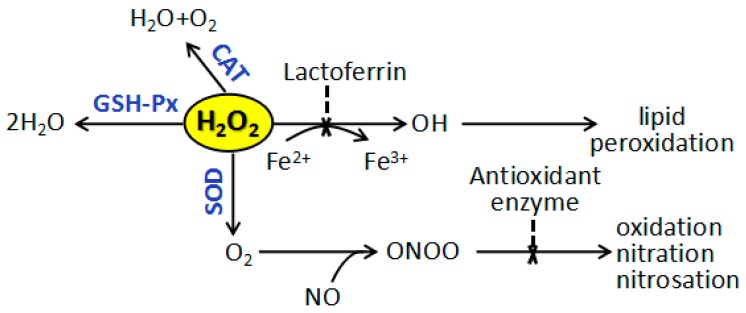
Antioxidant system of living organism.

The antioxidant defense system of living organisms is an organic whole and each pathway collaborates and enhances others. As shown in Figure 8, SOD, CAT, and GSH-Px are involved in the deletion of H_2_O_2_ to play a key role in preparing antioxidant protection to an organism. 

SOD deals with superoxide radical which can destroy GSH-Px and CAT, with the production of H_2_O_2_. Then in turn, H_2_O_2_ reduces cupricin the active center of SOD and lead to the enzyme inactivation. However, GSH-Px and CAT can remove H_2_O_2_ and protect SOD from oxidation. Moreover, GSH would replace CAT to eliminate H_2_O_2_ once CAT content appears low in some tissues. The functions of all these enzymes are interrelated and their reduced activities lead to the accumulation of lipid peroxides and enhancement oxidative stress. Treatment of 5′-AMP increased the activity levels of antioxidant enzymes (including SOD, CAT, and GSH-Px) and the amount of GSH, and decreased the level of MDA, thus decreased the accumulation of free radicals. It is suggested that 5′-AMP may protect the radiated body from oxidative damage by dynamically adjusting the REDOX balance.

## 4. Materials and Methods

### 4.1. Experimental Agents

Experimental agents were sourced from the following locations: 5′-AMP (Kaisheng Limited Co., Nanjing, China); RPMI-1640 (HyClone, Thermo Scientific Co., Waltham, MA, USA); fetal bovine serum (FBS, Southern American); anti-CD3-phycoerythrin (PE), anti-CD4-FITC, anti-CD8-FITC (BD Biosciences, Franklin Lakes, NJ, USA); interleukin-2 (IL-2), interleukin-4 (IL-4), interleukin-10 (IL-10), and interferon-γ (IFN-γ) enzyme-linked immunosorbent assay (ELISA) kits (NeoBioscience Technology Co., Ltd., Shenzhen, China); ConA (Sigma-Aldrich Co., St. Louis, MO, USA).

### 4.2. Experimental Animals

120 normal male KunMing mice (6~7 weeks old, 20~22 g) were obtained from the Harbin Veterinary Research Institute. The animals were housed under specific pathogen-free conditions. The animal room was controlled for temperature (22~24 °C), light (12 h light/dark cycles), and humidity (50~60%). Standard laboratory diet and tap water were supplied throughout the experiments. The animal ethical committee approval number is SCXK (Hei) 2006-009.

### 4.3. Experimental Design

After acclimation, the mice were randomized into six experimental groups (20 mice/group), as follows:

Group I: Normal mice (Normal group)
Group II: Radiation (Model group)
Group III: Radiation + berberine hydrochloride (Positive control group)
Group IV: Radiation + 5′-AMP (0.08 g/kgBW/day)
Group V: Radiation + 5′-AMP (0.16 g/kgBW/day)
Group VI: Radiation + 5′-AMP (0.64 g/kgBW/day)

The animals in Group I received no treatment (drug or radiation). The animals in Group II received 4 Gy whole-body γ-irradiation alone. The animals in Group III were administered berberine hydrochloride (20 mg/kgBW/day), as well as Groups IV~VI were administered 5′-AMP at doses of 0.08, 0.16, and 0.64 g/kgBW/day respectively, prior to irradiation. 5′-AMP and berberine hydrochloride were administered to the mice by an oral probe in their water suspension. After two weeks of drug treatment, the animals received 4 Gy whole-body ^60^Co γ-ray at the Institute of Application of Atomic Energy, Heilongjiang Academy of Agricultural Sciences, Harbin, China. The tested animals were irradiated at an acute single dose of 4 Gy delivered at 95 cm source-to-skin distance. The dose rate was 1.0 Gy/min. The dose was determined based on the relative researches [28,29] and our preliminary experiment. Mice were then sacrificed 1 day after exposure.

Spleen samples were immediately surgically removed, and weighed in a sterile hood. The spleen indexes were calculated using the following formula:

(1) $$Index(\%)=\frac{Weight_{spleen}}{Weight_{mousebody}}\times 100\%.$$

Four spleen samples were selected randomly from every group. One part of each spleen sample was removed and fixed in 2.5% (*v*/*v*) glutaraldehyde (pH 7.2) for examination by electron microscopy, and the remainder was used to measure total DNA content. The other spleen samples were used to isolate lymphocytes for cells cycle, apoptosis, cells subset, morphology, and cytokine assays.

### 4.4. Electron Microscopy Examination of Spleens in Mice

The fresh tissues from spleens were cut into sections of approximate 1 mm^3^ on parchment paper (based on wax plate). They were then prefixed in a solution of 2.5% (*v*/*v*) glutaraldehyde with phosphate buffer (pH 7.2) for 1 week at 4 °C. After rinsing three times in cold phosphate buffer (pH 7.2), the specimens were post fixed in 2% (*w*/*v*) osmium tetroxide for 1.5 h at 4 °C. Fixed tissues were then dehydrated in a series of graded ethanol (50%, 70%, 90% and 100%) for 8 min each time. Subsequently, the specimens were embedded in epoxy resin overnight. Ultrathin sections of approximate 50–70 nm were obtained with an ULTRACUT-E ultra microtome and stained with uranyl acetate and lead citrate for 15 min, respectively. The ultrathin sections were examined by an H-600 transmission electron microscope (Toshiba Corporation, Tokyo, Japan).

### 4.5. Measurement of Total DNA Contents in Mouse Spleen

DNA contents were assessed using the diphenylamine method. A standard curve was established based on a 200 μg/mL standard DNA solution (DNA sodium salt from calf thymus (Shanghai Invitrogen Biotechnology Co., Ltd., Shanghai, China) dissolved in 0.01 mol/L NaOH solution). DNA standard solutions containing 0, 0.4, 0.8, 1.2, 1.6 and 2.0 mL were added to six tubes, and the volumes were adjusted to 2 mL using distilled water. Diphenylamine (4.0 mL; 1 g recrystallized diphenylamine was dissolved in 100 mL glacial acetic acid mixed with 10 mL perchloric acid, which was added to 1.0 mL 2% acetaldehyde solution before use) was added to each test tube. Tubes were then shaken evenly, incubated at 90 °C for 15 min, and cooled. OD595 values were measured using a UV755 B spectrometer.

A section of the spleen (~25 mg) was homogenized in 5 mL of 0.01 mol/L NaOH solution, and centrifuged for 10 min at 4000 rpm to separate the supernatant. Four milliliters of diphenylamine was then added to 2 mL sample supernatant, which was then shaken, incubated at 90 °C for 15 min, and cooled. The OD 595 values were then measured as above. Distilled water instead of sample was used as a reference. The DNA contents in the sample were calculated based on the standard curve. The results were expressed as DNA in spleen tissue (μg/mg), and reactions were performed in triplicate.

(2) $${DNA}_{tissue}(\mu g/\mathrm{mg})=\frac{{DNA}_{solution}(\mu g)}{TissueWeight_{solution}(\mathrm{mg})}\times 2.5$$

### 4.6. Isolation of Lymphocytes from the Spleens of Mice

After the mice were sacrificed, spleens harvested under aseptic conditions were ground into small pieces and passed through sterilized meshes (200 meshes) to prepare crude splenocyte suspensions at room temperature. Samples were then centrifuged at 1000 rpm for 8 min at 4 °C, and the remaining splenocyte suspension was re-suspended using red blood cell lysis solution (Beyotime Institute of Biotechnology, Shanghai, China) to lyse the red blood cells. After a 1 min treatment, splenocyte suspensions were replenished using RPMI-1640 medium, and then centrifuged at 1000 rpm for 8 min at 4 °C. The pelleted splenocytes in each group were washed twice, and adjusted to concentrations of 2 × 10^6^ cells/mL with RPMI-1640 containing 10% FBS [30].

### 4.7. Analysis of Splenocyte Cell Cycle

Recovered splenocytes were fixed with pre-cooled 70% ethanol (−20 °C), and stored at 4 °C for 12 h. The fixative was removed carefully by centrifugation at 1000 rpm for 3 min. The pelleted splenocytes were re-suspended in 1 mL pre-cooled phosphate buffer solution (PBS) (0.01 mol/L, pH 7.2) at 4 °C after washing twice. The splenocyte suspensions were then labeled with propidium iodide (PI; PI/RNase Staining Buffer) for 1 h, and cells were analyzed by flow cytometry after filtering through a 0.4 μm membrane [31].

### 4.8. Analysis of Splenocyte Apoptosis

An FITC-Annexin-V/PI Double Staining Apoptosis Detection Kit (Biosea, Beijing, China) was used following the manufacturer’s instructions. Splenocyte suspensions were diluted to 5 × 10^6^ cells/mL in 200 μL binding buffer. FITC-Annexin V (10 μL) was added to the cell suspension, which was then incubated at room temperature for 15 min in the dark. Next, 300 μL of binding buffer and PI (propidium iodide, 5 μL) was added, and the cell suspensions were examined by flow cytometry within 1 h. A total of 10,000 cells per sample were analyzed using dual lasers at wavelengths of 525 nm and 575 nm.

### 4.9. Measurement of Cytokine Concentrations in the Supernatants of Lymphocytes

Splenocyte suspensions were prepared as described in Section 4.6 and adjusted to 2 × 10^7^ cells/mL. Then the cells were immediately plated into 24-well flat-bottom plates (0.5 mL per well). Treated with ConA at a final concentration of 5 μg/mL to induce cytokine secretion, cells were cultured for 72 h at 37 °C in a humidified incubator containing 5% CO_2_ at 37 °C. The supernatant from each well was harvested, and cytokine levels (IFN-γ, IL-2, IL-4 and IL-10) were measured using mouse ELISA kits (NeoBioscience, Shenzhen, China) according to the manufacturer’s instructions. Absorbances were measured at 450 nm using a microplate reader (Bio-Rad, Tokyo, Japan), and cytokine concentrations were calculated from a standard curve.

### 4.10. Analysis of Splenocyte Subsets

Phenotypic analyses of splenocyte suspensions were performed using double fluorescein staining. Splenocytes were incubated with monoclonal antibodies against CD3 and CD4 conjugated with different fluorochromes anti-CD3 (phycoerythrin (PE) and FITC, respectively), or CD3 and CD8 (conjugated to PE and FITC, respectively) in the dark for 15 min. The percentages of CD3^+^CD4^+^ and CD3^+^CD8^+^ splenocyte subgroups were then analyzed using flow cytometry (FACS Calibur BD Biosciences). Splenocyte suspensions stained with single CD3-PE, CD4-FITC, and CD8-FITC were used to adjust the instrumental compensation.

### 4.11. Determination of the T-SOD, GSH-Px, GSH, and MDA

After mice were sacrificed, the spleen tissues were immediately surgically removed, and the weighed spleen was homogenated with a certain volume of normal saline in an ice bath and made 10% spleen homogenates. After centrifugation (4000 rpm × 10 min) at 4 °C, the level of T-SOD, GSH-Px, GSH and MDA in spleen supernatant was determined according to experimental procedure provided by manufacturers (Nanjing Jiancheng Bioengineering Institute, Nanjing, China). Protein content was estimated according to the kit (BCA method) using BSA as standard.

### 4.12. Statistical Analysis

Data were analyzed using the Statistical Package for Social Science (version 19.0; SPSS Inc., IBM, Armonk, NY, USA) and reported as mean ± SD. Significant differences between groups were analyzed using one-way ANOVA. Significant differences were designated * *p* < 0.05 and ** *p* < 0.01 compared with the normal group, ^Δ^ *p* < 0.05 and ^ΔΔ^ *p* < 0.01 compared with the model group and ^▲^ *p* < 0.05 and ^▲▲^ *p* < 0.01 compared with positive control group.

## 5. Conclusions

**Figure 9:**
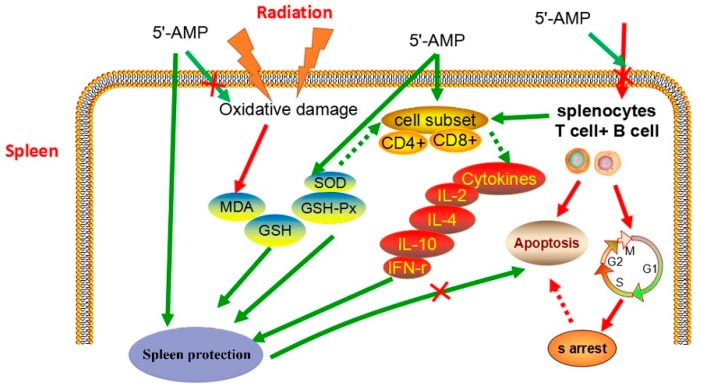
Proposed model for protective mechanism of 5′-AMP on spleen tissues of γ-ray irradiated mice.

In summary, this study suggested that 5′-AMP protected against radiation by enhancing the immune response. As described previously, Figure 9 shows that the mechanism for the beneficial effects of 5′-AMP on the immune system could involve: (1) regulating the cell cycle and apoptosis in lymphocytes; (2) the activation of T lymphocytes (CD4^+^ and CD8^+^); (3) increasing the production of various cytokines, including IFN-γ, IL-2, IL-4, and IL-10; and (4) increasing the level of antioxidant enzymes (including SOD, CAT, and GSH-Px) and the contents of GSH contents, and reducing MDA level.

## Figures and Tables

**Table 1:** Effects of adenosine 5′-monophosphate (5′-AMP) on the spleen organ index of mice treated with γ-radiation (*n* = 6).

Group	Spleen Index
Group I: Normal group	0.4869 ± 0.1659
Group II: Model group	0.1431 ± 0.0368 **
Group III: Positive control group	0.2214 ± 0.0556 **^,^^Δ^
Group IV: 5′-AMP (0.08 g/kgBW/day)	0.1754 ± 0.0332 **
Group V: 5′-AMP (0.16 g/kgBW/day)	0.2244 ± 0.0933 **^,^^Δ^
Group VI: 5′-AMP (0.64 g/kgBW/day)	0.1906 ± 0.0251 **

** *p* < 0.01 compared with the normal group; ^Δ^ *p* < 0.05 compared with the model group.

## Supplementary Materials

The following are available online at http://www.mdpi.com/1422-0067/19/5/1273/s1.
