# Supplementary material for: Protection of Spleen Tissue of γ-ray Irradiated Mice against Immunosuppressive and Oxidative Effects of Radiation by Adenosine 5′-Monophosphate
Source: Int J Mol Sci. 2018 Apr 24;19(5):1273. doi: 10.3390/ijms19051273 (PMC5983786; doi:10.3390/ijms19051273)
Supplement: Supplementary file 1 [file ijms-19-01273-s001.pdf]

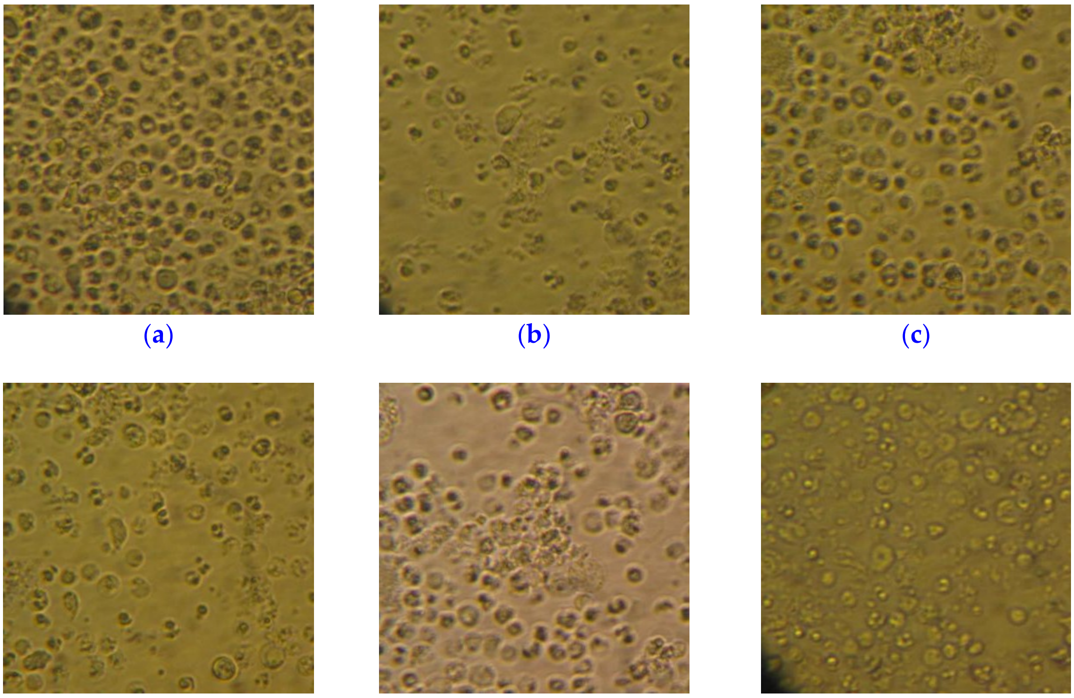

Figure S1. Morphology of splenocytes from different groups of mice after γ-ray irradiation (40×). (A) Group I: normal group; (B) Group II: model group; (C) Group III: positive control (berberine hydrochloride); (D) Group IV: Radiation + 5′-AMP (0.08 g/kg bw/day); (E) Group V: Radiation + 5′-AMP (0.16 g/kg bw/day); (F) Group VI: Radiation + 5′-AMP (0.64 g/kgbw/day).
